# The spatial dimension in biological data mining

**DOI:** 10.1186/1756-0381-4-6

**Published:** 2011-04-10

**Authors:** Davnah Urbach, Jason H Moore

**Affiliations:** 1 Dartmouth College, Institute for Quantitative Biomedical Sciences, One Medical Center Dr., Lebanon, NH 03756, USA; 2 Dartmouth Medical School, Department of Genetics, One Medical Center Dr., Lebanon, NH 03756, USA; 3 Dartmouth Medical School, Department of Community and Family Medicine, One Medical Center Dr., Lebanon, NH 03756, USA

## 

The beginning of the 21^st ^century has witnessed the generation of spectacular amounts of new information, ranging from marketing data to genomic sequences. As traditional statistical methods are gradually being defeated by both the amount of data and the general absence of underlying hypotheses, data mining procedures are becoming increasingly popular and user-friendly. By combining statistical-, artificial intelligence- and database management tools, those methods are tailored for processing large quantities of information and extracting interesting patterns. Since their first application, data mining procedures have progressively been tweaked to accommodate various types of information, including social science- and biological data. However, a number of features characteristic of biological data, including high levels of measurement variability and correlation between variables, represent an additional challenge and call for specific methods. The goal of this editorial is to highlight the spatial dimension of biological data mining.

Among its numerous applications, data mining plays an increasingly important role in epidemiology. In particular, it allows processing the steadily increasing volume of genomic data and helps identifying genetic risk factors. Despite ongoing progress, the mining methods currently manufactured for exploring such data still stumble over their very characteristic features and in particular their considerable complexity and diversity. Genomic data range from DNA sequences and single nucleotide polymorphisms (SNPs) to gene and protein expression levels and protein-protein interaction patterns, and further encompass structural and functional genome annotation. Accordingly, various types of data are generally treated independently and patterns emerging from any set of analyses are stitched together to form a biological answer or to generate new hypotheses.

Occasionally, such patterns are projected onto a geographical map, superimposed to migration patterns or correlated to environmental factors, placing crude numeric information into a spatio-temporal perspective [reviewed in [1]]. Integrating spatial, environmental and genetic data into models of geographic disease etiology (ecogeographic genetic epidemiology) has recently been proposed as a new interdisciplinary pathway to understand the distribution and the determinants of diseases [1]. The Geographic Information Systems (GIS) used to integrate these multiple layers of information is a set of powerful hardware and software for inputting, managing, displaying and analyzing geographically referenced information. GIS have relatively recently been recognized as a useful tool for biomedical research, and in particular for visualizing cancer distributions and estimating the contribution of various environmental risk factors to cancer prevalence [reviewed in [1]]. Accordingly, the American National Cancer Institute http://gis.cancer.gov/, with the Long Island Breast Cancer Study Project for instance http://li-gis.cancer.gov/, has a long-lasting research program investigating geographic and environmental patterns of cancer using GIS technologies. Other initiatives such as cancer atlases (e.g. http://www.cdc.gov/Features/CancerAtlas/) testify to the utility of spatial analyses in epidemiology.

GIS-based approaches have so far proven useful in retrospectively identifying spatial diseases patterns or environmental factors likely to contribute to the expression of genetically determined diseases. However, GIS technologies are still underexploited and underrated in biomedical sciences. Here we suggest that there is room for creative minds to imagine innovative ways to apply these techniques and to merge them with data mining procedures. For example, prior to engaging into a genome-wide association study and testing vast numbers of SNPs independently for a correlation with disease susceptibility, geo-referenced population-wide SNP data could be superimposed to maps of disease rates to identify subsets of candidate disease-associated SNPs. Subsequent case-control studies could then be performed on those subsets to corroborate or refute the hypothesis of a true association. The spatial dimension of biological data in epidemiology and other areas such as image analysis provides unique challenges and opportunities for biological data mining.
